# Investigation on Physico Chemical and X-ray Shielding Performance of Zinc Doped Nano-WO_3_ Epoxy Composite for Light Weight Lead Free Aprons

**DOI:** 10.3390/ma16103866

**Published:** 2023-05-20

**Authors:** Sanjeevi Palanisami, Vishnu Shankar Dhandapani, Varuna Jayachandran, Elango Muniappan, Dongkyou Park, Byungki Kim, Kalpana Govindasami

**Affiliations:** 1 Department of Physics, PSG College of Arts & Science, Coimbatore 641014, India; 2 Department of Electromechanical Convergence Engineering, Korea University of Technology and Education, Cheonan 31253, Republic of Korea; 3 School of Mechatronics Engineering, Korea University of Technology and Education, Cheonan 31253, Republic of Korea; 4 Department of Science and Humanities, Tamilnadu College of Engineering, Coimbatore 641659, India

**Keywords:** Zn/WO_3_ nanoparticles, epoxy-nano composite, lead free aprons, physical characterization, X-ray attenuation analysis

## Abstract

This report addresses a way to reduce the usage of highly toxic lead in diagnostic X-ray shielding by developing a cost-effective, eco-friendly nano-tungsten trioxide (WO_3_) epoxy composite for low-weight aprons. Zinc (Zn)-doped WO_3_ nanoparticles of 20 to 400 nm were synthesized by an inexpensive and scalable chemical acid–precipitation method. The prepared nanoparticles were subjected to X-ray diffraction, Raman spectroscopy, UV-visible spectroscopy, photoluminescence, high-resolution–transmission electron microscope, scanning electron microscope, and the results showed that doping plays a critical role in influencing the physico-chemical properties. The prepared nanoparticles were used as shielding material in this study, which were dispersed in a non-water soluble durable epoxy resin polymer matrix and the dispersed materials were coated over a rexine cloth using the drop-casting method. The X-ray shielding performance was evaluated by estimating the linear attenuation coefficient (μ), mass attenuation coefficient (μ_m_), half value layer (HVL), and X-ray percentage of attenuation. Overall, an improvement in X-ray attenuation in the range of 40–100 kVp was observed for the undoped WO_3_ nanoparticles and Zn-doped WO_3_ nanoparticles, which was nearly equal to lead oxide-based aprons (reference material). At 40 kVp, the percentage of attenuation of 2% Zn doped WO_3_ was 97% which was better than that of other prepared aprons. This study proves that 2% Zn doped WO_3_ epoxy composite yields a better particle size distribution, μ_m_, and lower HVL value and hence it can be a convenient lead free X-ray shielding apron.

## 1. Introduction

It is known that numerous clinical personnel are exposed to diagnostic radiation every day [1]. Exposure to radiation at a certain level causes serious issues for human beings. Long-term exposure to radiation above the safety level or without appropriate productive gear may cause serious and fatal effects such as cancer, cell damage, and other undesirable health issues [2]. Lead (Pb) is the traditional material used in aprons which acts as radiation shielding material for clinical operators due to its high atomic density and high X-ray attenuation co-efficient [3]. However, Pb poses a serious threat to human health, especially infants and it is very difficult to recycle and store. The incorporation of lead oxide nanoparticles onto a polymer matrix creates a nanocomposite material that is more robust and requires less maintenance. Consequently, the content of lead used in shielding material would lessen. The polymer matrix acts as a protective layer around the nanoparticles, shielding them from external stresses. Some researchers have found that Pb-based dimethyl polysiloxane, polystyrene and polystyrene-b-polyethyleneglycol composite materials yield better attenuation than pure Pb [4,5,6,7]. As a result of the European Union’s prohibition of the use of Pb in European healthcare, we increased the urgency of replacing Pb-based aprons with those made of alternative materials [1]. So, along with Pb, other materials such as bismuth and tungsten related compounds were investigated as radiation shielding material due to their high atomic number (z) [8]. Among them, tungsten is a material of choice with a high X-ray attenuation coefficient, high k-absorption edge, and the lowest degrees of toxicity [8]. In addition, tungsten oxide is a much safer material and more delicate to use in diagnostic radiation aprons [9]. However, to improve the chemical and physical stability, tungsten derivatives such as tungsten oxide can be considered as an alternative material for effective X-ray shielding. This material is inexpensive, easily viable and includes other advantages such as low toxicity, recyclability and safe storage capability compared with toxic Pb. Considering all these factors that are to be prioritized for humans, tungsten oxide would be the best choice to replace toxic lead.

In the ideology of lead-free aprons, polyvinyl alcohol (PVA)-based micro and nanocomposites have been used in X-ray and gamma ray shielding in recent times. Studies have used PVA blended molybdenum nanoparticles, tin oxide, silver chloride and tungsten trioxide in X-ray and gamma ray blocking materials and the results are acceptable [10,11]. Similarly, there is a sustainable effect observed on electrospun PVA-based nanofibers filled with bismuth oxide and tungsten trioxide as a radiation shielding material [9]. In other polymers such as polyethylene, nature rubber, isophthalic resin, poly dimethyl siloxane, polyboron, epoxy resin, methylsilsesquioxane, and fiber plastic [12]. At present, researchers are focusing on lead-free metal oxides polymer composites such as polymethyl-methacrylate matrix filled with bismuth oxide (Bi_2_O_3_), bismuth (Bi) and tungsten (W) composite, Bi_2_O_3_, tungsten trioxide (WO_3_), tungsten carbide (W_2_C), tantalum pentoxide (Ta_2_O_5_) filled with polypropylene prepared by three-dimensional needled shielding fabric and epoxy doped zinc oxide (ZnO) [13,14,15,16]. The epoxy resin was chosen in this work as an alternative dispersion medium owing to it is readily available and easily scalable [17]. The epoxy resin is thermosetting polymer structure-based epoxy which is cross-linked to form a highly rigid and durable network structure [18]. It is also durable compared to other polymers with high mechanical properties, easy processing with low shrinkage during the casting process [19]. In addition to that, epoxy is a thermoplastic which can withstand higher temperatures, making it a suitable material for power washing [19]. For the final part of the preparation of aprons, a thin nylon supported rexine cloth was used to increase its reusability with low wear and tear [20]. The rexine and epoxy combination offer an excellent adhesion to one another with a strong binding tendency [21]. Compared to the other fibers, the low porosity of rexine offers a good sealing property which also protects the epoxy—nanoparticle composites and prevents from the damage of skin by blocking the radiations.

Using micro-particles instead of nanoparticles with the epoxy as a compound layer results in less homogeneous, void development in the aprons [22]. By reducing the particle size and shrinking them, we can address this problem [23]. Therefore, innovative lead-free radiation protection materials can be created using the special doped nanomaterials characteristics associated with nanomaterials [24,25]. The size lowered nanoparticles especially based lead-free aprons exhibit good scattering and improved absorption of secondary X-rays [26]. The addition of a dopant can change the effective atomic number (Zeff) of the composite material, which can affect its X-ray shielding properties. Hence, in this work, we used nanoparticles of Zn-doped WO_3_ as dispersion, epoxy as a composite layer, and nylon supported rexine as a base layer for the fabrication of the apron. The variation in the concentration of Zn (1%, 2%, 3%) in the WO_3_ matrix alters the physical properties of WO_3_ [27], which are studied for the application of X-ray shielding in this work.

## 2. Materials and Methods

### 2.1. Synthesis of Zn-WO_3_ Nanoparticles

WO_3_ nanoparticles were prepared through the acid-precipitation method [28]. First, sodium tungstate dihydrate (Na_2_WO_4_·2H_2_O) was dissolved in 600 mL of double distilled water. In this solution, 0.0, 1.0, 2.0, and 3.0 mol% of zinc chloride (ZnCl_2_) was added, and hydrochloric acid (HCl) was added dropwise until the pH of the medium turns 1. After adding HCl, the solution was turned yellow in color which indicated the formation of tungstic acid (H_2_WO_4_). Then it was allowed to age for 24 h at room temperature under normal stirring and subjected to sonication. The precipitate was collected by centrifugation and washed with the help of distilled water to remove sodium chloride (NaCl), which was shown in the chemical reaction (R2). The precipitate was calcinated at 500 °C and H_2_O got evaporated, which was given by the chemical reaction (R2) [29].

(R1) $${\mathrm{Na}}_{2}{\mathrm{WO}}_{4}\cdot 2H_{2}O+2\mathrm{HCl}+H_{2}O\;\to H_{2}{\mathrm{WO}}_{4}+2\mathrm{NaCl}+3H_{2}O$$

(R2) $$H_{2}{\mathrm{WO}}_{4}+3H_{2}O\overset{∆}{\to }{\mathrm{WO}}_{3}+4H_{2}O{↑}_{\left(g\right)}$$

Finally, the calcinated product was ground for further characterization and apron fabrication. The 0.0, 1.0, 2.0, and 3.0% Zn doped samples were labeled as WZ0, WZ1, WZ2, and WZ3 respectively hereafter.

### 2.2. Fabrication of Zn-WO_3_ Nanocomposite Apron and Measurement Set-Up

**Figure 1:**
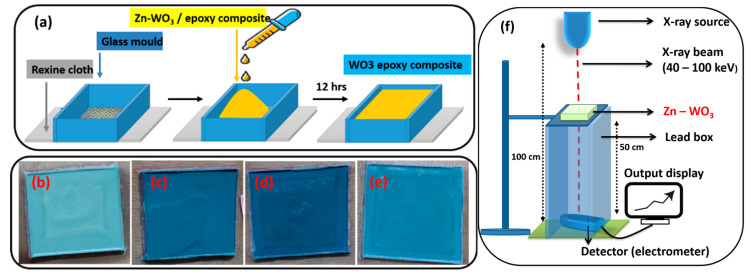
(**a**) Schematic representation of the fabrication process. (**b**–**e**) Prepared WZ0, WZ1, WZ2, and WZ3 epoxy nanocomposite-based aprons used for X-ray shielding studies. (**f**) Graphical depiction of X-ray attenuation study.

The filler (prepared nanoparticles) and epoxy polymers were mixed in a 1:1 ratio weight percentage using a magnetic stirrer and uniformly dispersed. A glass cuboid template with a dimension of 4 cm × 4 cm was placed over a rexine cloth. The nanocomposite was spread using the drop-casting method over the rexine cloth to mimic the fabric of the apron. It was allowed to rest for 12 h at room temperature and the desired study material was acquired. This preparation process for the apron is shown in Figure 1a,b, respectively.

A sample size of 4 cm × 4 cm was kept over a sample holder made up of lead with an opening of 3 cm × 3 cm at the center. The sample and detector were located at 50 cm and 100 cm respectively from the X-ray source [30]. A lead box was placed behind the sample enclosing the detector to prevent the scattering of X-rays. The exposure was set at 10 mAs, time was set at 2 s and the X-ray tube voltage was between 40 and 100 kVp. An X-ray beam of 1 cm × 1 cm width from the X-ray source was allowed to pass through the sample. The penetrated radiation was received by the detector and read from the display.

### 2.3. Characterization

The structural properties were identified using the model Empyrean, Malvern Panalytical Powder X-ray Diffraction (XRD) instrument (Malvern, UK) using the Cu K_α_ source (λ = 0.154 nm). The optical properties of prepared samples were analyzed through UV-visible (UV-Vis) optical spectrometer using the model Jasco V770, 28600 mary’s court Easton, MD, USA 21601. The defect and local structural properties were confirmed and analyzed through photoluminescence (PL) and Raman scattering studies using the perkins Elmer LS45 (Ulm, Germany), and WiTec alpha 300 (355 and 532 nm laser) (Ulm, Germany) respectively. The surface morphology, elemental analysis, and elemental mapping were carried out by EVO 18 model’s scanning electron microscope (SEM) (Jena, Germany) and energy dispersive X-ray spectroscopy (EDX) (Jena, Germany) studies respectively. The fine structure such as lattice planes, grain size, and selected area diffraction (SAED) was carried out by high-resolution transmission electron microscope (HR-TEM) model JEOL JEM 2100 (Peabody, MA, USA). For X-ray attenuation studies, M/S Wipro Ge Healthcare’s fixed radiography model HF (Banglore, India)advantage was used for producing a 40–100 kV X-ray source with an average exposure time of 2 s and the source to image distance (SID) of 100 cm [30,31] detected using the RTI Piranha 557 electrometer (Molndal, Swedan).

## 3. Results

### 3.1. XRD Analysis

**Figure 2:**
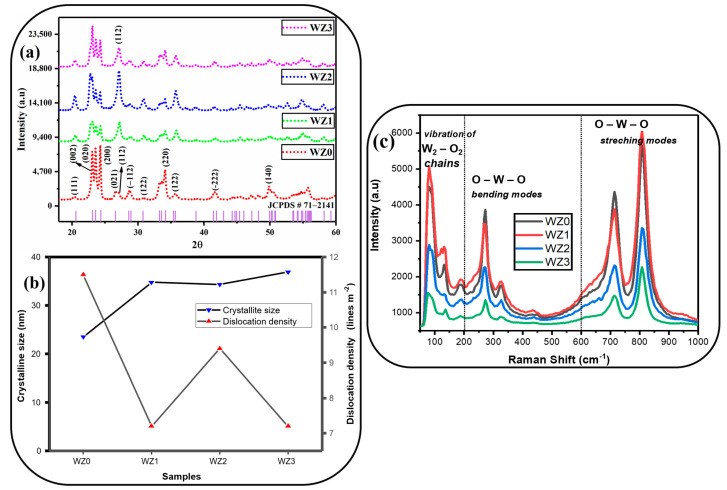
(**a**) XRD spectra of undoped and Zn doped WO_3_ nanoparticles. (**b**) Crystallite size Vs dislocation density correlation. (**c**) Raman spectra of WZ0, WZ1, WZ2, WZ3 nanoparticles.

XRD analysis was performed to investigate the crystalline size, crystal structure and lattice characteristics of Zn-doped WO_3_ nanopowders. Table 1 shows the crystalline structure of prepared nanoparticles. Figure 2a shows the X-ray diffraction patterns of pure WO_3_ and Zn-doped WO_3_ nanopowders annealed at 500 °C. The XRD result shows major intense peaks at 23.1°, 23.5°, 24.3°, 26.6°, 33.2°, 33.5°, 34.1° and 49.9° which correspond to the planes of (002), (020), (200), (120), (022), (−202), (202) and (140) respectively. These planes match well with the JCPDS card No: 71-2141 and confirm the formation of monoclinic WO_3_ particles [32]. The prominent peak of WZ0 is located at (200) direction. The direction of the prominent peak changed when the dopant is introduced. Zn prefers to locate in the (120) direction for the samples WZ1 and WZ2. WZ3 peak is located at the (002) direction. For pure WO_3_ particles, the lattice parameters have been calculated as a = 0.732 nm, b = 0.750 nm, and c = 0.768 nm from Equation (1) [33], which was also in good agreement with the standard data. Upon doping, the peak positions remain the same with negligible shift but the intensity changes with respect to the dopant concentration since the zero presence of additional peaks belong to elemental or compound impurities, which shows that there is no secondary phase formation or elemental impurity presence in the prepared samples. The crystallite size (D), cell volume (v), dislocation density (δ) and the micro strain (ԑ) are calculated using Equations (1)–(5) [33,34].

(1) $$\frac{1}{d^{2}}=\frac{1}{\sin^{2}\beta }\left(\frac{h^{2}}{a^{2}}+\frac{k^{2}\sin^{2}\beta }{b^{2}}+\frac{l^{2}}{c^{2}}-\frac{2\mathrm{hl}\;\cos\beta }{\mathrm{ac}}\right)$$

(2) $$D=\frac{0.9\lambda }{\beta \cos\theta }$$

where, d—interplanar distance, a, b, c—lattice parameters, hkl—miller indices, β—full width half maximum (FWHM) and k—Scherrer constant.

(3) V=abc sinβ

(4) $$\delta =\frac{1}{D^{2}}$$

(5) $$\varepsilon =\frac{\lambda }{D\;\sin\theta }-\frac{\beta }{\tan\theta }$$

where, θ—peak position and λ—X-ray wavelength (Cu k_α_ = 0.1514 nm).

The changes in lattice parameters are due to different concentrations of doping and are given in Table 1. This is due to the incorporation of Zn ions into the WO_3_ lattice matrix where it alters the crystalline properties of the WO_3_ nanoparticles [35]. From the XRD result, it is evident that doping causes the structural parameters to change. Especially the sharp increase in intensity of the peak (120) shows less crystallite size for 2% doping, owing to the smaller ionic radii of Zn^2+^ (0.074 nm) ions compared to W^6+^ ions (0.078 nm) [27]. Causes the charge imbalance in lattice planes and alters the interplanar distance. This change in interplanar distance causes other parameters to deviate, resulting in an improved crystallite size. For increased Zn concentrations, crystallite size is found to be slightly greater than 2% but smaller when compared to a pure sample. Among all the samples, WZ2 exhibits better crystalline quality than other prepared materials. Figure 2b shows the crystallite size vs dislocation density trend for various Zn dopant concentrations in the WO_3_ matrix. Using the formula below, we are able to determine the stacking fault (SF) and texture coefficient (TC) [36,37].

(6) $$\mathrm{SF}=\frac{2\pi^{2}}{45{\left(\tan\theta \right)}^{1/2}}\;\beta$$

(7) $${\;\mathrm{TC}}_{\left(\mathrm{hkl}\right)}=\frac{\left[I_{\left(\mathrm{hkl}\right)}/I_{0\left(\mathrm{hkl}\right)}\right]}{ƩN[I_{\left(\mathrm{hkl}\right)}/I_{0\left(\mathrm{hkl}\right)}]}\times 100\%$$

where, N is the number of diffraction peaks, I_(hkl)_ and I_0(hkl)_ are the measured relative intensity of a plane and the standard intensity of the plane, taken from JCPDS data. The TC value is varied for different doping concentrations. The sample WZ2 has the highest TC value which indicates that it has plenty of closely oriented crystallites and the lowest TC value of the sample WZ3 represents that it possesses randomly oriented crystallites. The fluctuation of SF is caused by the formation of crystal defects. The presence of SF in a crystal produces a shift in the phase of incident and scattered X-rays with regard to the lattice, altering the consequent peak location. As WZ0 has the highest crystalline size, SF is low. When dopant is introduced, the crystalline size is reduced resulting in an increase in SF. Among the samples, WZ2 is associated with the highest SF value (Table 1). These properties of WZ2 make it a better X-ray attenuation material.

### 3.2. Raman Analysis

Figure 2c shows the Raman spectra of prepared pure WO_3_ and Zn-doped WO_3_ (WZ0, WZ1, WZ2, WZ3) nanoparticles. Table 2 shows the Raman spectra peak position of prepared nanoparticles. The pure WO_3_ particles exhibit intense peaks at 81, 131, 188, 272, 326, 713, and 807 cm^−1^. In general, the observed peaks from 200 to 500 cm^−1^ and from 600 to 1000 cm^−1^ represent the O-W-O bending modes and W-O stretching modes of the WO_3_ system respectively. Peaks below 200 cm^−1^ are attributed to the translational or rotational lattice modes in WO_6_ octahedron [32,38]. Among these, the peaks at 81 and 131 cm^−1^ correspond to the vibration of W_2_O_2_ chains which is a unique mode of the monoclinic phase of WO_3_ nanoparticles [32]. The peaks at 713 and 808 cm^−1^ represent the stretching vibration of O-W-O ions [39]. This is in good agreement with the XRD results and confirms the formation of monoclinic WO_3_ particles. The peaks at 272 and 326 cm^−1^ reveal the plane bending vibration of the O-W-O ions [39]. Upon doping of Zn ions into a lattice, the peak positions show negligible shifts and a sharp change in intensity. The negligible peak position shift may originate from several factors such as a laser-induced heating effect or morphological changes in the focusing point [34]. The observed results reveal the complete dissolution of Zn ions into the WO_3_ lattices. The change in the Raman peak intensity is ascertained by Zn doping effect.

### 3.3. Optical Properties

**Figure 3:**
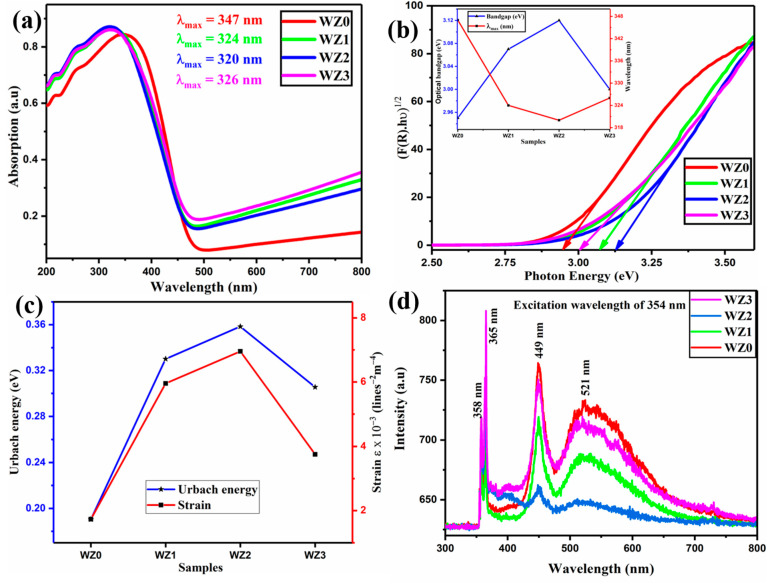
(**a**) UV-Vis absorption spectra of synthesized WZ0, WZ1, WZ2, and WZ3 nanoparticles. (**b**) Kubelka–Munk plot for the synthesized nanoparticles (Figure insert: Variation in bandgap vs. maximum absorption wavelength). (**c**) Urbach energy Vs strain plot. (**d**) PL spectra of synthesized nanoparticles of undoped and Zn doped WO_3_ (WZ0, WZ1, WZ2, WZ3) system.

The optical properties of pure WO_3_ and Zn-doped WO_3_ nanoparticles were explored using UV-Vis-DRS spectra. The DRS spectra of the prepared nanoparticles manifest strong band edge absorption in the UV-Vis region. All the samples exhibit their band edge absorption between 320 to 345 nm (shown in Figure 3a), which might be due to the intermolecular charge transfer or to the conjugation system [40]. The sharp absorption peak between 320 to 345 nm shows the well crystalline quality of all the samples [41,42]. All the Zn-doped WO_3_ nanoparticles are blue-shifted when compared to the WZ0 sample, which confirms that the optical properties of WO_3_ nanoparticles are highly influenced by Zn doping [43]. Using the Kubelka–Munk plot (Figure 3b), the optical bandgap is estimated to be 2.88 eV at room temperature for WZ0. The optical bandgap energy of doped samples namely WZ1, WZ2, and WZ3 is higher than that of the pure WZ0 nanosystem, the energy gap values of the samples are calculated as 3.01 eV, 3.09 eV, and 2.97 eV respectively. This result reveals that the Burstein–Moss effect plays a major role [44]. The doping of semiconductor materials moves the fermi level beyond the conduction band due to the formation of a larger number of donor levels [45]. Hence the bandgap obtained from such doped material is found to be enlarged. For the doped oxide nanoparticles, the optical bandgap energy variation is owing to the synergetic effect betwixt Zn and WO_3_ [33,43]. The values are consistent with the reported value [46]. Urbach energy (E_u_) is known as the width of the defect bands, which are produced by the charge disproportionation, and creates an impact on the optical transitions betwixt the valance and conduction bands as well. These defect bands introduced the Urbach tail or localised states in band tails. E_u_ is the term for the energy connected to this defect tail. These defects may also have developed throughout the development process, leading to lattice abnormalities and stress in the sample. The E_u_ has been calculated by taking the inverse of the slope of the equation [47].

ln α = ln α_0_ + hυ/E_u_ (8)

The creation of defect levels in betwixt the band gaps obviously enhances the E_u_ with Zn dopant. A higher E_u_ shows that the sample WZ2 is more prone to transform weak bonds into defects [48]. This is also consistent with the strain behaviour reported in XRD studies (Figure 3c).

The defect properties of prepared nanoparticles were investigated using photoluminescence (PL) spectra at room temperature. The excitation wavelength of the PL spectrum is 355 nm. The WO_3_ nanoparticles at various dopant concentration levels exhibit three emission bands as shown in Figure 3c. The PL spectra for both WO_3_ and Zn-doped WO_3_ nanoparticles showcase similar emission bands and dopants do give or raise any new peaks from PL phenomena. However, the intensity of dopant materials (WZ1, WZ2, WZ3) is found to be reduced compared to WZ0. The sharp and high-intensity peak centred at 365 nm (3.39 eV) corresponds to the ultraviolet (UV) band. It is due to the recombination of electrons from the internal states of the charged oxygen vacancies in the conduction band to the valence band [49,50]. WZ3 material has a strong emission band at the wavelength of 365 nm (3.39 eV) originating from higher content of surface oxygen vacancy. Due to this vacancy, the WZ3 material has less crystallinity compared with WZ0 [33,51]. The second emission band was observed as blue emission of 449 nm (2.76 eV) which may be attributed to the presence of radiative recombination betwixt the bandgap energy of 2.76 eV or oxygen vacancy defect in WO_3_ [52,53]. The third emission band for the samples WZ0, WZ1, WZ2, and WZ3 are located at 522, 517, 513, and 521 nm respectively. The low intense and broad peak is related to green band emission caused by the presence of interstitial oxygen vacancy. The oxygen defects or vacancy gives rise to the green colour for WO_3_ nanoparticles rather than its native yellow colour [54]. While increasing the Zn dopant concentration from WZ1 to WZ2 the intensity of the green emission peak decreases and shifts to a lower wavelength. Further increasing the Zn concentration, the intensity of the green emission becomes raised, and position of the peak is shifted to a higher wavelength region. This phenomenon is ascertained by the deep defect level present in the material WZ3 [33,55]. It is noteworthy that, after dispersing the WO_3_ nanoparticles in the epoxy resin, the colour of the WO_3_ changes much dark and turns bluish-grey in colour. The change in colour is assumed as epoxy reacts with WO_3_ nanoparticles and there may be a bond formation between epoxy and WO_3_ nanoparticles. This will give rise to strong structural integrity.

### 3.4. HR-TEM and SAED Analysis

**Figure 4:**
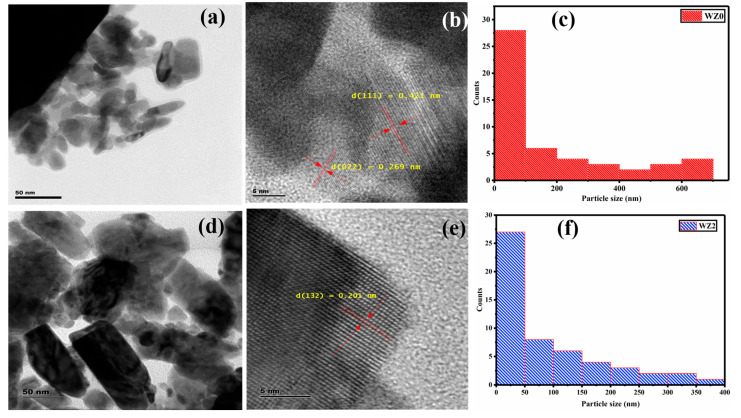
(**a**,**b**,**d**,**e**) TEM micrographs of WZ0 and WZ2 nanoparticles. (**c**,**f**) Histogram of particle size distribution.

Figure 4a shows the TEM image of WZ0nanoparticles with agglomerated pseudo-cuboid morphology which may become clustered together during the growth and annealing process. The larger particles are anchored by the smaller particles on the surface, such agglomeration and clustering are common limitations in the solution-grown nanoparticles [28]. The particle size distribution plot for Figure 4a shown in Figure 4c conveys the poly-dispersed state of the nanoparticles. i.e., the size of the larger particles was in the range of 300–700 nm and the size of smaller particles adhered on the surface of the larger particle was in the range of 20–100 nm. This smaller particle anchoring on the larger particle can be seen clearly in Figure 4a. The TEM micrograph of WZ2 nanoparticles showcased the distinct and agglomeration-free state. The doping of Zn into WO_3_ reduces the overall size of the nanoparticles (Figure 4d) compared to WZ0. The average size distribution of the particles in the WZ2 sample is found to be 10–50 nm. It is also witnessed that medium-size particles are in the range from 60–250 nm and with aggregated particles are in the range of 300–400 nm (Figure 4f). Also, upon doping, the morphology of the particles transformed from cuboid shape to cube and rods with uneven edges. The WZ2 shows well-defined lattice fringes in Figure 4e. The incorporation of Zn into the WO_3_ monoclinic system orients the crystallites into a single direction and limits the poly crystallization of WO_3_. Also, the 2% Zn doping improves the crystalline quality of the particle which is in good agreement with the XRD analysis findings.

**Figure 5:**
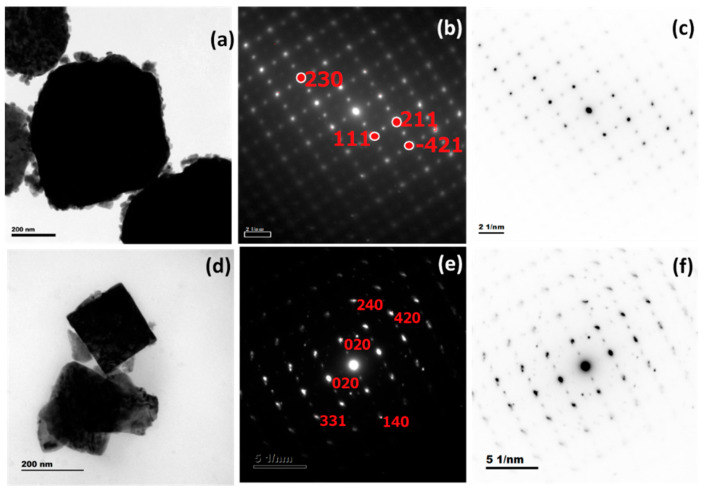
(**a**,**d**) TEM micrographs of WZ0 and WZ2 samples. (**b**,**e**) hkl indexed dot pattern of WZ0 and WZ2 samples. (**c**,**f**) Inverse contrast applied the SAED pattern of WZ0 and WZ2, respectively.

Figure 5a–f shows the SAED pattern images of undoped WO_3_ and 2% Zn doped WO_3_ nanoparticles obtained from HR-TEM. The inverse contrast image of the SAED pattern clearly distinguishes the pure and doped WO_3_. In the pure WO_3_, the dot patterns are arranged precisely with high periodicity, on the other hand, the doped one exhibits less distorted spots. The observation clearly demonstrates the differences in the lattice property of the Zn-doped lattice from the pure WO_3_. Overall, the result proves the formation of monoclinic WO_3_ and the incorporation of Zn ions into the WO_3_ lattice does not alter the monoclinic structure of WO_3_.

### 3.5. SEM-EDAX Analysis

**Figure 6:**
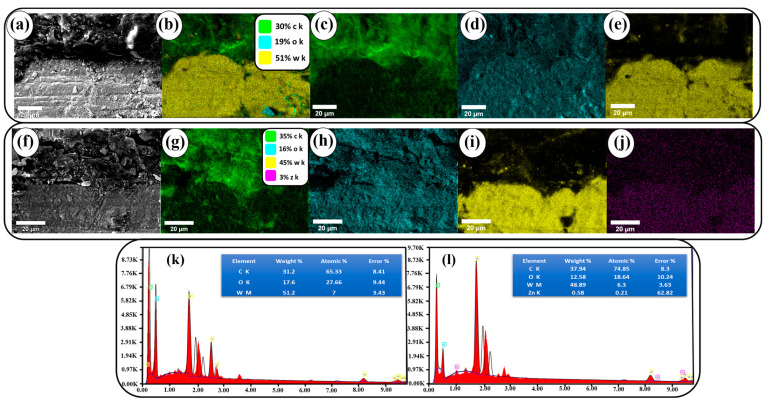
(**a**–**j**) SEM mapping of WZ0 and WZ2 apron cross-section. (**k**,**l**) EDS spectra and the weight composition of WZ0 and WZ2 samples.

The cross-sectional view of WZ0 and WZ2 impregnated epoxy-resin aprons was subjected to SEM and elemental mapping analysis and the results are shown in Figure 6a–j. The presence of carbon in the elemental mapping is reasoned for the rexine cloth. Zn, W, O are also witnessed in the study which confirms the formation of compound Zn doped WO_3_. Figure 6h–j confirms the even distribution of Zn, W, and O elements in the apron in the form of Zn-doped WO_3_ nanoparticles.

Figure 6k,l shows the EDS spectra of WZ0 distributed apron and WZ2 impregnated epoxy-rexine apron. The spectra show the presence of carbon, tungsten, and oxygen. The peak of carbon is originated from the rexine and epoxy. Zn, W and O peaks in the spectra confirmed that Zn is doped in the WO_3_ lattice. The apron with a low concentration of Zn exhibits a less intense peak. The other small peaks presented in EDS are attributed due to the rexine and epoxy compound materials.

### 3.6. X-ray Shielding Analysis

**Figure 7:**
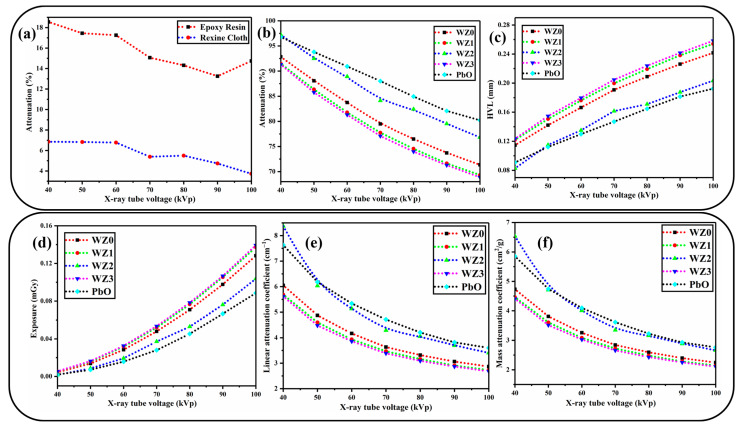
(**a**) X-ray attenuation percentage of pure epoxy and rexine cloth. (**b**–**f**) explain the attenuation percentage, HVL, Exposure, μ and μ_m_ analysis of WZ0, WZ1, WZ2, WZ3 and PbO-based aprons respectively.

Much experimental evidence for the interaction of X-ray photons with matter are available in literature starting from Thomson scattering to Compton scattering. Many researchers have realised that their works reveal either absorbed (or) scattered (or) attenuated matter. In the energy range, below 200 kVp, the photoelectric effect (PEE) is the predominant phenomenon of X-rays that is accompanied by characteristic radiation, photoelectrons, and positive ions. Above 200 kVp, Compton scattering comes into existence with X-ray photons [56]. In the present study, employed diagnostic X-rays are in the energy range of 20–140 kVp which is primarily used in the angiographic, orthopaedic and dental X-ray imaging processes [33]. To estimate the innate attenuation of X-rays by the rexine and epoxy resin, X-ray attenuation studies were performed on the pure epoxy coated over a rexine cloth. The results show that (Figure 7a,b), the epoxy rexine layers have linear attenuation throughout the 40–100 kVp range. The epoxy shows higher X-ray attenuation than the bare rexine cloth. The prepared WZ0, WZ1, WZ2, and WZ3 nanoparticles are separately dispersed uniformly in the epoxy resin under vigorous stirring and coated over the rexine cloth via the drop-casting method. Though a wide variety of aprons are commercially available in market, by following the same experimental protocol we prepare a PbO impregnated epoxy resin–rexine cloth apron as a reference material. The X-ray attenuation percentage is evaluated from the relation below [31]

(9) $$\%\mathrm{Attenuation}=\frac{\;\mathrm{Electrometer}\;\mathrm{reading}\;\mathrm{without}\;\mathrm{sample}-\mathrm{Electrometer}\;\mathrm{reading}\;\mathrm{with}\;\mathrm{sample}}{\mathrm{Electrometer}\;\mathrm{reading}\;\mathrm{without}\;\mathrm{sample}}\times 100\%$$

**Figure 8:**
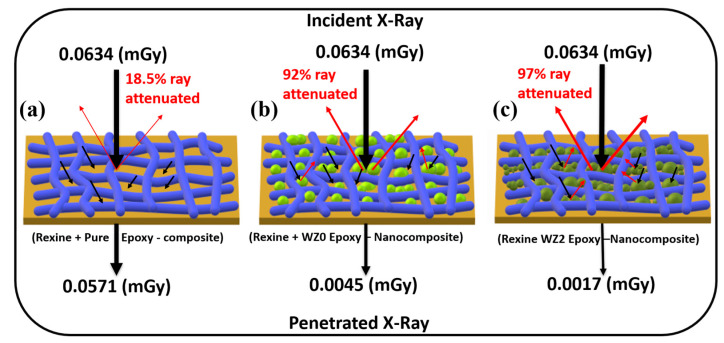
Schematic diagram of X-ray attenuating property for (**a**) Rexine and pure epoxy composite, (**b**) Rexine and WZ0 epoxy nanocomposite, (**c**) Rexine and WZ2 epoxy nanocomposite.

The WZ0, WZ1, WZ2, and WZ3 nanoparticles-based apron and the PbO-based apron show similar trends in X-ray attenuation performance over the entire test range. This trend is maintained in the X-ray attenuation at higher tube voltages, especially between 70 and 100 kVp. A slight increase in the attenuation value of a Pb-based apron is attributed to its high atomic number (Pb = 82) compared to tungsten (W = 74), but the difference in X-ray attenuation between the prepared aprons is less significant in real-world operation [8]. Also, contrary to expectation, among the Zn-doped WO_3_ system-based apron, WZ2 performed well in the X-ray attenuation compared to pure and other dopant concentrations (WZ0, WZ1 and WZ3). The X-ray attenuation of WZ2 is better than that of PbO for lower tube voltage (40–50 kVp). Besides, Half-Value-Layer measurements are also made for the prepared aprons, the numerical value of the HVL is found using the relation (10) and (11), which is also an important parameter to estimate the X-ray attenuating apron [8]. It is also interesting to notice that HVL is the thickness of the sample adequate enough to reduce the initial intensity of the X-rays into half [57,58].

(10) $$\mathrm{HVL}=\frac{0.693}{\mu }$$

(11) $$\mu =-\frac{1}{t}\mathrm{Ln}\left(\frac{I}{I_{o}}\right)$$

(12) $$\mu_{m}=\frac{\mu }{\rho }$$

(13) $$\rho =\frac{m1}{m1-m2}\rho_{l}$$

where, µ–Liner attenuation coefficient, t—thickness of the sample, I—intensity of attenuated beam and I_o_—initial intensity, μ_m_ —mass attenuation coefficient, ρ—density of the material (density of unknown materials was calculated by the Archimedes method), m1—mass in air, m2—mass in immersion liquid and ρ_l_ is the density of the immersion liquid (here, ethanol is immersion liquid ρ_l_ = 0.789 g/cm^−1^). From the results (Figure 7c), WZ2 showed a low HVL value for a low energy range of 40–50 kVp of tube voltage. Beyond which, it is found that the value of HVL increases linearly throughout the voltage range. The prepared aprons WZ0, WZ1 and WZ3 also showed a similar trend of increasing HVL values. All the samples exhibit a higher value than the Pb based apron. The sample WZ2 shows low HVL for the energy range 40–50 kVp and starts to increase exponentially for the energy range 50–100 kVp. It is noteworthy that, the value of the HVL of WZ2 based apron is higher for than the HVL value of PbO based apron in the higher energy range (80–100 kVp). Figure 7d represents the tube voltage vs exposure, which provides the information about quality of radiation reaching the detector after passing through the apron. It is understood that PbO and WZ2 have identical and lowest exposure among all the samples. After which the second lowest exposure is exhibited by WZ0 followed by WZ1- and WZ3-based aprons. The important reason behind the similar exposure of WZ2 and PbO in X-ray shielding is that the size effect of Zn-doped WO_3_ nanoparticles compared to the micro-sized PbO particles in an apron. The uniform distribution of nanoparticles in the epoxy matrix is also attributed to similar exposure behaviour of PbO-based apron and WZ2-based apron [26]. Linear attenuation coefficient (μ) is calculated by Equation (11). If the thickness (t) of the material varies, μ might vary in the same material. It means, μ depends on the t of the given material. In the present study t of samples was not changed and Figure 7e shows the μ value for prepared WZ0, WZ1, WZ2, and WZ3 based aprons and PbO-based apron. At low tube voltage (40–50 kVp) WZ2 has a high μ value. As tube voltage increases further (50–100 kVp), μ of PbO was slightly more than that of WZ2 since in this energy region, photoelectric effect absorption is high for higher atomic number (Pb = 82) [59]. Also, μ of WZ2 is better than WZ0, WZ1, and WZ3. By dividing the μ by density (ρ), the resultant coefficient is ρ independent and is known as the mass attenuation coefficient (μ_m_). The μ_m_ and ρ of the aprons are calculated by Equations (12) and (13), the ρ tabulated in Table 3. Figure 7f shows that WZ2 based apron had the highest μ_m_ value in lower tube voltage (40–50 kVp) and PbO marginally high in the higher tube voltage (50 kV–100 kVp). μ_m_ values of WZ0, WZ1 and WZ3 based aprons are lower than WZ2 based apron. Due to the photoelectric effect, the attenuation value dropped as photon energy increased in the low energy range (40–100 kVp) [60]. The comparison of μ_m_ between the prepared sample and other polymer metal oxide composites and alloy materials is tabulated in Table 4. Figure 8 shows the reason for the better X-ray shielding property of WZ2 based apron than other aprons. Since there were no materials to block the X-ray, there was only 25% of the incident X-rays are attenuated (Figure 8a). The presence of pure WO_3_ in the apron (Figure 8b) increases the attenuation up to 92%. There were no sufficient smaller-size particles in the pure WO_3_ to block the X-rays. However, 2% Zn-doped WO_3_ (Figure 8c) shows increased attenuation of 97% which is reasoned as a high amount of smaller- sized particles (10–50 nm) of WZ2 in WZ2 based aprons which seal the space between the large-sized nanoparticles and epoxy resin fibers. Our report proves that doping Zn in a WO_3_ matrix can alter the X-ray attenuation property of a material. The reduced particle size with improved crystallinity (obtained from TEM and XRD) of WZ2 also supports the above argument. The smaller the particle, the higher the grain boundary scattering probability coupled with the secondary scattering events, since, the number of particles per gram is greater compared to the lead particles [61,62,63].

Further, we presumed that the high surface to volume ratio of the nanosystem shows enhanced X-ray attenuation properties, since a greater number of WO_3_ (or) Zn-doped WO_3_ system are exposed themselves to incoming X-ray radiation. This phenomenon also greatly increases the chance of absorbing the X-ray photon which ends up with the blocking of X-rays. Thus, nanoparticle impregnation, in an either undoped (or) doped form improves the X-ray attenuation coefficient than (or) equal to PbO-based apron. There are several types of tungsten-based polymer composites that can be used for X-ray shielding; Table 4 compares their X-ray attenuation performance. Only minimal studies have been conducted on tungsten-based epoxy nanocomposite. In this study, Zn-doped WO3-based epoxy nanocomposite material is used for the first time to the best of my knowledge in an X-ray shielding application and the results are compared to other materials. Results show that the addition of a dopant enhanced the X-ray attenuation performance.

## 4. Conclusions

The X-ray shielding properties of Zn-doped WO_3_ composite aprons were studied and discussed in detail. The pure and Zn-doped WO_3_ nanoparticles were prepared using the acid preparation method. The XRD results show the crystalline nature with a monoclinic structure and reduction in crystalline size on the addition of Zn dopant, Raman vibration modes also confirmed the monoclinic structure. The optical study of UV-visble spectroscopy showed bandgap changes due to Zn doping. The addition of a dopant creates a defect in the parent material, which is shown by the photoluminescence study. TEM image revealed that the particle size of 2% Zn-doped WO_3_ was smaller than undoped WO_3_ and smaller particles in the 2% Zn-doped WO_3_ were anchored to the larger particles, creating a better X-ray attenuation property by sealing the space between the large particles. The HVL of the 2% Zn-doped WO_3_ system revealed less value and hence, it exhibited better attenuation in low-energy region. The study revealed that the WZ2-based apron had good mass attenuation coefficient (μ_m_), which made it as an efficient material in X-ray shielding process. The results of the study demonstrate that doping can affect the X-ray attenuation efficiency of a material and that Zn doped WO_3_ can be used as an alternative to lead for environmentally friendly and durable X-ray shielding applications.

## Figures and Tables

**Table 1:** Crystalline properties of WZ0, WZ1, WZ2 and WZ3 nanomaterials.

Sample	Lattice Parameters (Å)			Cell Volume (Å^3^)	Crystallite Size (nm)	Dislocation Density (10^15^) Lines m^−2^	Micro Strain (Ԑ) × 10^−3^ Lines^−2^ m^−4^	Texture Coefficient (TC_(hkl)_)	Stacking Fault
	a	b	c						
WZ0	7.32	7.50	7.68	422	29.2	1.19	1.73	1.08	0.37
WZ1	7.27	7.56	7.70	424	12.9	5.96	2.65	1.58	0.75
WZ2	7.29	7.47	7.72	421	11.9	6.95	2.86	2.08	0.82
WZ3	7.28	7.48	7.71	420	16.3	3.75	2.09	0.63	0.70

**Table 2:** Raman spectra peak analysis of Zn doped WO_3_ particles (WZ0, WZ1, WZ2, WZ3).

Samples	Peak Position (cm^−1^)						
WZ0	81.8	131.8	188.8	272.3	326.8	713.5	807.7
WZ1	80.8	131.5	188.3	270.4	326.3	713.7	807.7
WZ2	80.8	133	190.7	270.4	326.8	715.9	809.7
WZ3	76.5	136	186.7	272.5	326.3	713.7	807.7

**Table 3:** Composite Thickness and density of prepared aprons.

Samples (with Epoxy Composite)	Thickness (10^−3^ m)	Density (g/cm^3^)
WZO	4.36	1.29
WZ1	4.33	1.27
WZ2	4.29	1.28
WZ3	4.35	1.27
PbO (reference material)	4.50	1.30
Pure epoxy (Polymer)	4.15	1.05

**Table 4:** X-ray attenuation performance comparison between tungsten oxide and different polymer composites shielding materials.

Material	Density (g/cm^3^)	X-ray Energy (kVp)	Major Results	Reference
SRM-50 (dimethyl polysiloxane + wt 50% PbO)	2.038	59.51 and 80.99	HVL ≈ 2 and 4	[4]
SRN-50 (dimethyl polysiloxane + wt 50% PbO)	2.086		HVL ≈ 2.5 and 3	
Portland Cement (89%) + PU-Plaster (1%) + Lead Oxide (10%)	-	121.78–1408.01	HVL ≈ 30 and 125	[5]
PS/PbO (A) 35	1.4015	40–100	μ = 9.918 − 1.062	[7]
PS/PbO (B) 35	1.3560		μ = 9.829 − 1.440	
B5 [(10% PVA) (WO_3_ Filler loading 40%)]	≈1.29	8.64–25.20, 57.53	μ_m_ ≈ 155 − 25.20, 130	[9]
EPVC/micro WO_3_ 40 wt%	-	40 and 100	μ_m_ = 11.20 and 5.49 HVL = 0.5 and 0.9	[8]
EPVC/nano WO_3_ 40 wt%			μ_m_ = 11.81 and 5.68 HVL = 0.5 and 1	
WP1(W/polymer ration 300%)	-	150	μ_m_ ≈ 3.5	[64]
Micro WO_3_/E44 & Nano WO_3_/E44	2.18, 2.29	59.6	μ_m_ = 1.0196, 1.0706	[65]
W-SR (W wt 80%)	-	59.5	μ_m_ ≈ 2.05	[66]
W0.115at%	-	40–100	μ_m_ ≈ 0.34 − 0.24	[19]
PE-A-25 (PE 75%, TBG 25%) (TBG − nano WO_3_ + BI_2_O_3_ + GO)	1.43	50–120	μ_m_ = 0.26 − 0.15	[67]
Wt 40% WO_3_ + PVA	1.79	5.89–59.54	μ_m_ = 127.60 − 1.33	[68]
W	4.18	50 and 120	μ_m_ = 8.74 and 5.25	[69]
Sn-W	4.2		μ_m_ = 9.89 and 5.71	
W-Ba	3.11		μ_m_ = 9.51 and 5.55	
PVA + wt 40% WO_3_ (micro)	-	^201^Tl-radioactivesources	μ_m_ ≈ 0.98 HVL = 3	[70]
PVA + wt 40% WO_3_ (nano)			μ_m_ ≈ 1.28 HVL = 2	
WN400m	≈1.9	60–120	μ_m_ ≈ 14.2 − 8	[71]
Epoxy + wt 50% PbO	1.30	40–100	μ_m_ = 5.85 − 2.76 HVL = 0.09 − 0.19	Our work
Epoxy + wt 50% WZ0 (undoped WO_3_ NPs)	1.29		μ_m_ = 4.73 − 2.24 HVL = 0.12 − 0.24	
Epoxy + wt 50% WZ2 (2% Zn doped WO_3_)	1.28		μ_m_ = 6.54 − 2.66 HVL = 0.08 − 0.21	

Units: HVL = mm, μ = cm^−1^, μ_m_ = cm^2^ g^−1.^

## Data Availability

Not applicable.

## References

[1] Maghrabi H.A., Vijayan A., Deb P., Wang L. (2016). Bismuth Oxide-Coated Fabrics for X-ray Shielding. Text. Res. J..

[2] Le Heron J., Padovani R., Smith I., Czarwinski R. (2010). Radiation Protection of Medical Staff. Eur. J. Radiol..

[3] Özdemir T., Güngör A., Akbay I.K., Uzun H., Babucçuoglu Y. (2018). Nano Lead Oxide and Epdm Composite for Development of Polymer Based Radiation Shielding Material: Gamma Irradiation and Attenuation Tests. Radiat. Phys. Chem..

[4] El-Khatib A.M., Abbas M.I., Hammoury S.I., Gouda M.M., Zard K., Elsafi M. (2022). Effect of PbO-Nanoparticles on Dimethyl Polysiloxane for Use in Radiation Shielding Applications. Sci. Rep..

[5] Cinan Z.M., Baskan T., Erol B., Mutlu S., Misirlioglu Y., Yilmaz S.S., Yilmaz A.H. (2021). Gamma Irradiation, Thermal Conductivity, and Phase Change Tests of the Cement-Hyperbranched Poly Amino-Ester-Block-Poly Cabrolactone-Polyurathane Plaster-Lead Oxide and Arsenic Oxide Composite for Development of Radiation Shielding Material. Int. J. Energy Res..

[6] Cinan Z.M., Erol B., Baskan T., Mutlu S., Yilmaz S.S., Yilmaz A.H. (2021). Gamma Irradiation and the Radiation Shielding Characteristics: For the Lead Oxide Doped the Crosslinked Polystyrene-b-Polyethyleneglycol Block Copolymers and the Polystyrene-b-Polyethyleneglycol-Boron Nitride Nanocomposites. Polymers.

[7] Osman A.F., El Balaa H., El Samad O., Awad R., Badawi M.S. (2023). Assessment of X-ray Shielding Properties of Polystyrene Incorporated with Different Nano-Sizes of PbO. Radiat. Environ. Biophys..

[8] Asari Shik N., Gholamzadeh L. (2018). X-ray Shielding Performance of the EPVC Composites with Micro- or Nanoparticles of WO_3_, PbO or Bi_2_O_3_. Appl. Radiat. Isot..

[9] Jamil M., Hazlan M.H., Ramli R.M., Noor Azman N.Z. (2019). Study of Electrospun PVA-Based Concentrations Nanofibre Filled with Bi_2_O_3_ or WO_3_ as Potential X-ray Shielding Material. Radiat. Phys. Chem..

[10] Kawady N.A., Elkattan M., Salah M., Galhoum A.A. (2022). Fabrication, Characterization, and Gamma ray Shielding Properties of PVA-Based Polymer Nanocomposite. J. Mater. Sci..

[11] Abulyazied D.E., Saudi H.A., Zakaly H.M.H., Issa S.A.M., Henaish A.M.A. (2022). Novel Nanocomposites Based on Polyvinyl Alcohol and Molybdenum Nanoparticles for Gamma Irradiation Shielding. Opt. Laser Technol..

[12] More C.V., Alsayed Z., Badawi M.S., Thabet A.A., Pawar P.P. (2021). Polymeric Composite Materials for Radiation Shielding: A Review.

[13] Wang B., Qiu T., Yuan L., Fang Q., Wang X., Guo X., Zhang D., Lai C., Wang Q., Liu Y. (2023). A Comparative Study between Pure Bismuth/Tungsten and the Bismuth Tungsten Oxide for Flexible Shielding of Gamma/X rays. Radiat. Phys. Chem..

[14] Wang W., Liu Y., Li S., Dong K., Wang S., Cai P., Hou L., Dou H., Liang D., Algadi H. (2023). Lead-Free and Wearing Comfort 3D Composite Fiber-Needled Fabric for Highly Efficient X-ray Shielding. Adv. Compos. Hybrid Mater..

[15] Alsaab A.H., Zeghib S. (2023). Study of Prepared Lead-Free Polymer Nanocomposites for X- and Gamma-ray Shielding in Healthcare Applications. Polymers.

[16] Mahmoud K.G., Tashlykov O.L., Praveenkumar S., Sayyed M.I., Hashim S. (2023). Synthesis of a New Epoxy Resin Reinforced by ZnO Nanoparticles for γ-ray Shielding Purposes: Experimental and Monte Carlo Simulation Assesments. Radiat. Phys. Chem..

[17] La L.B.T., Leatherday C., Leong Y.K., Watts H.P., Zhang L.C. (2018). Green Lightweight Lead-Free Gd_2_O_3_/Epoxy Nanocomposites with Outstanding X-ray Attenuation Performance. Compos. Sci. Technol..

[18] Kausar A. (2017). Role of Thermosetting Polymer in Structural Composite. Kausar A Am. J. Polym. Sci. Eng..

[19] Noor Azman N.Z., Siddiqui S.A., Ionescu M., Low I.M. (2012). Synthesis and Characterisation of Ion-Implanted Epoxy Composites for X-ray Shielding. Nucl. Instrum. Methods Phys. Res. Sect. B Beam Interact. Mater. Atoms.

[20] Hasan K.M.F., Wang H., Mahmud S., Jahid M.A., Islam M., Jin W., Genyang C. (2020). Colorful and Antibacterial Nylon Fabric via In-Situ Biosynthesis of Chitosan Mediated Nanosilver. J. Mater. Res. Technol..

[21] Meyer M., Dietrich S., Schulz H., Mondschein A. (2021). Comparison of the Technical Performance of Leather, Artificial Leather, and Trendy Alternatives. Coatings.

[22] Zhang X., Fan X., Yan C., Li H., Zhu Y., Li X., Yu L. (2012). Interfacial Microstructure and Properties of Carbon Fiber Composites Modified with Graphene Oxide. ACS Appl. Mater. Interfaces.

[23] Lopresti M., Palin L., Alberto G., Cantamessa S., Milanesio M. (2021). Epoxy Resins Composites for X-ray Shielding Materials Additivated by Coated Barium Sulfate with Improved Dispersibility. Mater. Today Commun..

[24] Archer B.R. (2005). Recent History of the Shielding of Medical X-ray Imaging Facilities. Health Phys..

[25] Künzel R., Okuno E. (2012). Effects of the Particle Sizes and Concentrations on the X-ray Absorption by CuO Compounds. Appl. Radiat. Isot..

[26] Noor Azman N.Z., Musa N.F.L., Nik Ab Razak N.N.A., Ramli R.M., Mustafa I.S., Abdul Rahman A., Yahaya N.Z. (2016). Effect of Bi_2_O_3_ Particle Sizes and Addition of Starch into Bi_2_O_3_–PVA Composites for X-ray Shielding. Appl. Phys. A Mater. Sci. Process..

[27] Kalanur S.S., Noh Y.G., Seo H. (2020). Engineering Band Edge Properties of WO_3_ with Respect to Photoelectrochemical Water Splitting Potentials via a Generalized Doping Protocol of First-Row Transition Metal Ions. Appl. Surf. Sci..

[28] Nayak A.K., Ghosh R., Santra S., Guha P.K., Pradhan D. (2015). Hierarchical Nanostructured WO_3_-SnO_2_ for Selective Sensing of Volatile Organic Compounds. Nanoscale.

[29] Gong Q., Cen K., Yu C. (2003). Experimental Study on Supercritical Phenomena of WO_3_ Solubility in NaCl-H_2_O System. Sci. China Ser. D Earth Sci..

[30] Kim S.C., Choi J.R., Jeon B.K. (2016). Physical Analysis of the Shielding Capacity for a Lightweight Apron Designed for Shielding Low Intensity Scattering X-rays. Sci. Rep..

[31] Nambiar S., Osei E.K., Yeow J.T.W. (2013). Polymer Nanocomposite-Based Shielding against Diagnostic X-rays. J. Appl. Polym. Sci..

[32] Kumar V.B., Mohanta D. (2011). Formation of Nanoscale Tungsten Oxide Structures. Bull. Mater. Sci..

[33] Deepa B., Rajendran V. (2018). Pure and Cu Metal Doped WO_3_ Prepared via Co-Precipitation Method and Studies on Their Structural, Morphological, Electrochemical and Optical Properties. Nano-Struct. Nano-Objects.

[34] Karthick K., Kathirvel P., Marnadu R., Chakravarty S., Shkir M. (2021). Ultrafast One Step Direct Injection Flame Synthesis of Zinc Oxide Nanoparticles and Fabrication of P-Si/n-ZnO Photodiode and Characterization. Phys. B Condens. Matter.

[35] Cheng X.F., Leng W.H., Liu D.P., Zhang J.Q., Cao C.N. (2007). Enhanced Photoelectrocatalytic Performance of Zn-Doped WO_3_ Photocatalysts for Nitrite Ions Degradation under Visible Light. Chemosphere.

[36] Thilagavathi T., Venugopal D., Marnadu R., Chandrasekaran J., Alshahrani T., Shkir M. (2021). An Investigation on Microstructural, Morphological, Optical, Photoluminescence and Photocatalytic Activity of WO_3_ for Photocatalysis Applications: An Effect of Annealing. J. Inorg. Organomet. Polym. Mater..

[37] Singh G., Shrivastava S.B., Jain D., Pandya S., Shripathi T., Ganesan V. (2010). Effect of Indium Dop Ing on Zinc Oxide Films Prepared by Chemical Spray Pyrolysis Technique. Bull. Mater. Sci..

[38] Balaji S., Djaoued Y., Albert A.S., Brüning R., Beaudoin N., Robichaud J. (2011). Porous Orthorhombic Tungsten Oxide Thin Films: Synthesis, Characterization, and Application in Electrochromic and Photochromic Devices. J. Mater. Chem..

[39] Nonaka K., Takase A., Miyakawa K. (1993). Raman Spectra of Sol-Gel-Derived Tungsten Oxides. J. Mater. Sci. Lett..

[40] Nakahara H., Fukuda K. (1981). Electronic Spectra and Molecular Orientation in Multilayers of Long-Chain Anthraquinone Derivatives. J. Colloid Interface Sci..

[41] Wang H., Baek S., Lee J., Lim S. (2009). High Photocatalytic Activity of Silver-Loaded ZnO-SnO_2_ Coupled Catalysts. Chem. Eng. J..

[42] Height M.J., Pratsinis S.E., Mekasuwandumrong O., Praserthdam P. (2006). Ag-ZnO Catalysts for UV-Photodegradation of Methylene Blue. Appl. Catal. B Environ..

[43] Wang W.W., Zhu Y.J., Yang L.X. (2007). ZnO-SnO_2_ Hollow Spheres and Hierarchical Nanosheets: Hydrothermal Preparation, Formation Mechanism, and Photocatalytic Properties. Adv. Funct. Mater..

[44] Atheek P., Puviarasu P., Basha S.M., Bhujel K. (2022). Micro Raman Analysis on the Impact of Light Ion Irradiation of Hydride Vapor-Phase Epitaxy Grown Gallium Nitride Epilayers. Thin Solid Films.

[45] Svitasheva S.N., Gilinsky A.M. (2013). Influence of Doping Level on Shift of the Absorption Edge of Gallium Nitride Films (Burstein-Moss Effect). Appl. Surf. Sci..

[46] Matalkeh M., Nasrallah G.K., Shurrab F.M., Al-Absi E.S., Mohammed W., Elzatahry A., Saoud K.M. (2022). Visible light photocatalytic activity of Ag/WO3 nanoparticles and its antibacterial activity under ambient light and in the dark. Results Eng..

[47] Singh J., Verma V., Kumar R., Kumar R. (2019). Influence of Mg^2+^-Substitution on the Optical Band Gap Energy of Cr_2−x_Mg_2−x_O_3_ Nanoparticles. Results Phys..

[48] Mamat M.H., Sahdan M.Z., Amizam S., Rafaie H.A., Khusaimi Z., Rusop M. (2009). Optical and Electrical Properties of Aluminum Doped Zinc Oxide Thin Films at Various Doping Concentrations. J. Ceram. Soc. Japan.

[49] Luo J.Y., Xu N.S., Zhao F.L., Deng S.Z., Tao Y.T. (2011). Ultraviolet Superfluorescence from Oxygen Vacancies in WO_3−x_ Nanowires at Room Temperature. J. Appl. Phys..

[50] Cho H.D., Yoon I.T., Chung K.B., Kim D.Y., Kang T.W., Yuldashev S.U. (2018). Low-Temperature Photoluminescence of WO_3_ Nanoparticles. J. Lumin..

[51] Hirano M., Okamoto T. (2018). Synthesis, Morphology, and Luminescence of ZnNb_2_O_6_ Nanocrystals by Hydrothermal Method. Nano-Struct. Nano-Objects.

[52] Mohammadi S., Sohrabi M., Golikand A.N., Fakhri A. (2016). Preparation and Characterization of Zinc and Copper Co-Doped WO_3_ Nanoparticles: Application in Photocatalysis and Photobiology. J. Photochem. Photobiol. B Biol..

[53] Kavitha V.S., Reshmi Krishnan R., Sreeja Sreedharan R., Suresh K., Jayasankar C.K., Mahadevan Pillai V.P. (2019). Tb^3+^-Doped WO_3_ Thin Films: A Potential Candidate in White Light Emitting Devices. J. Alloys Compd..

[54] Bourdin M., Gaudon M., Weill F., Duttine M., Bourdin M., Gaudon M., Weill F., Duttine M., Gayot M., Bourdin M. (2020). Nanoparticles (NPs) of WO_3−x_ Compounds by Polyol Route with Enhanced Photochromic Properties. Nanomaterials.

[55] Wang F., Di Valentin C., Pacchioni G. (2011). Semiconductor-to-Metal Transition in WO_3−x_: Nature of the Oxygen Vacancy. Phys. Rev. B—Condens. Matter Mater. Phys..

[56] Peters S.M.B., Zweers D., de Lange F., Mourik J.E.M. (2017). Lead Composite vs. Nonlead Protective Garments: Which Are Better? A Multivendor Comparison. Radiat. Prot. Dosim..

[57] Reda A.M., El-Daly A.A. (2020). Gamma ray Shielding Characteristics of Sn-20Bi and Sn-20Bi-0.4Cu Lead-Free Alloys. Prog. Nucl. Energy.

[58] Spierings A.B., Schneider M., Eggenberger R. (2011). Comparison of Density Measurement Techniques for Additive Manufactured Metallic Parts. Rapid Prototyp. J..

[59] Buchtela K., Oesterreichischen A. (2005). Der Gamma ray Spectrometer. Anal. Chem..

[60] Issa S.A.M., Sayyed M.I., Zaid M.H.M., Matori K.A. (2018). Photon Parameters for Gamma-rays Sensing Properties of Some Oxide of Lanthanides. Results Phys..

[61] Li Q., Zhong R., Xiao X., Liao J., Liao X., Shi B. (2020). Lightweight and Flexible Bi@Bi-La Natural Leather Composites with Superb X-ray Radiation Shielding Performance and Low Secondary Radiation. ACS Appl. Mater. Interfaces.

[62] Mesbahi A., Ghiasi H. (2018). Shielding Properties of the Ordinary Concrete Loaded with Micro- and Nano-Particles against Neutron and Gamma Radiations. Appl. Radiat. Isot..

[63] Botelho M.Z., Künzel R., Okuno E., Levenhagen R.S., Basegio T., Bergmann C.P. (2011). X-ray Transmission through Nanostructured and Microstructured CuO Materials. Appl. Radiat. Isot..

[64] Kim Y., Park S., Seo Y. (2015). Enhanced X-ray Shielding Ability of Polymer-Nonleaded Metal Composites by Multilayer Structuring. Ind. Eng. Chem. Res..

[65] Dong Y., Chang S.Q., Zhang H.X., Ren C., Kang B., Dai M.Z., Dai Y.D. (2012). Effects of WO_3_ Particle Size in WO_3_/Epoxy Resin Radiation Shielding Material. Chinese Phys. Lett..

[66] Dejangah M., Ghojavand M., Poursalehi R., Gholipour P.R. (2019). X-ray Attenuation and Mechanical Properties of Tungsten-Silicone Rubber Nanocomposites. Mater. Res. Express.

[67] Abdolahzadeh T., Morshedian J., Ahmadi S. (2023). Novel Polyethylene/Tungsten Oxide/Bismuth Trioxide/Barium Sulfate/Graphene Oxide Nanocomposites for Shielding against X-ray Radiations. Int. J. Radiat. Res..

[68] Muthamma M.V., Gudennavar B.S., Gudennavar S.B. (2020). Attenuation Parameters of Polyvinyl Alcohol-Tungsten Oxide Composites at the Photon Energies 5.895, 6.490, 59.54 and 662 KeV. Polish J. Med. Phys. Eng..

[69] Morshedian J., Reza M., Darounkola R., Mansoori E., Keshvari R. (2023). New Flexible Non-Toxic X-ray Shielding Hybrid Materials Based on X-SBR Liquid Rubber. Radiat. Phys. Chem..

[70] Hosseini M.A., Malekie S., Kazemi F. (2022). Experimental Evaluation of Gamma Radiation Shielding Characteristics of Polyvinyl Alcohol/Tungsten Oxide Composite: A Comparison Study of Micro and Nano Sizes of the Fillers. Nucl. Instrum. Methods Phys. Res. Sect. A Accel. Spectrometers Detect. Assoc. Equip..

[71] Yun J., Hou J., Jang W., Kim S.Y., Byun H. (2022). Electrospun Tungsten-Polyurethane Composite Nanofiber Mats for Medical Radiation-Shielding Applications. ChemNanoMat.

